# Depression, Diabetes, and Chronic Disease Risk Factors Among US Women of Reproductive Age

**Published:** 2011-10-15

**Authors:** Sherry L. Farr, Donald K. Hayes, Rebecca H. Bitsko, Pooja Bansil, Patricia M. Dietz

**Affiliations:** Division of Reproductive Health, National Center for Chronic Disease Prevention and Health Promotion, Centers for Disease Control and Prevention; Centers for Disease Control and Prevention, Atlanta, Georgia; Centers for Disease Control and Prevention, Atlanta, Georgia; Centers for Disease Control and Prevention, Atlanta, Georgia; Centers for Disease Control and Prevention, Atlanta, Georgia

## Abstract

**Introduction:**

Depression and chronic disease have implications for women's overall health and future pregnancies. The objective of this study was to estimate the prevalence and predictors of diabetes and chronic disease risk factors among reproductive-age women with depression.

**Methods:**

We used population-based data from the 2006, 2008, and 2010 Behavioral Risk Factor Surveillance System to examine prevalence of diabetes and prediabetes, binge and heavy drinking, smoking, overweight and obesity, and physical inactivity among 69,043 women aged 18 to 44 years with current major or minor depression, a past depression diagnosis, or no depression. In a multivariable logistic regression model, we calculated adjusted odds ratios (AORs) and 95% confidence intervals (CIs) of 1, 2, and 3 or more chronic disease risk factors by depression status.

**Results:**

We found that 12.8% of reproductive-aged women experienced both current depression and 1 or more chronic disease risk factors. Compared to women with no depression, currently depressed women and those with a past diagnosis had higher prevalence of diabetes, smoking, binge or heavy drinking, obesity, and physical inactivity (*P* < .001 for all). Odds of 3 or more chronic conditions and risk factors were elevated among women with major (AOR, 5.7; 95% CI, 4.3-7.7), minor (AOR, 4.7; 95% CI, 3.7-6.1), and past diagnosis of depression (AOR, 2.8; 95% CI, 2.4-3.4).

**Conclusion:**

Depressed women of reproductive age have high rates of chronic disease risk factors, which may affect their overall health and future pregnancies.

## Introduction

Population-based data show that more than 14% of US women of reproductive age screen positive for major or minor depression (1). In the general population, depression is correlated with risk factors for chronic disease, such as smoking, excessive alcohol use, obesity, and physical inactivity (2). Independent associations also exist between depression and cardiovascular disease (3,4) and diabetes (5). Chronic disease comorbidities compromise the health of many people with depression; however, depression and chronic disease comorbidities among women of reproductive age have unique implications for the health of the woman and any future pregnancies.

Numerous studies confirm that behavioral and lifestyle risk factors, such as smoking, excessive alcohol consumption, and overweight and obesity, may adversely affect pregnancy outcomes. Depression may also negatively affect pregnancy outcomes (6). Poor mental health, including depression, may be a barrier to lifestyle changes in other areas, and incorporating mental health counseling into behavioral change interventions may be necessary for success (7,8). Thus, understanding the comorbidity of depression and chronic disease risk factors among women of reproductive age may benefit chronic disease prevention and treatment strategies. In a previous analysis of 2006 Behavioral Risk Factor Surveillance System (BRFSS) data, we examined prevalence of depression, associated demographic characteristics, and access to care among women of reproductive age (1). The objective of this analysis was to examine population-based estimates of the burden of diabetes and chronic disease risk factors among reproductive-age women with depression.

## Methods

We analyzed data from the 2006, 2008, and 2010 BRFSS, a state-based random-digit–dialed survey of the noninstitutionalized US population aged 18 years or older. In 2006, 38 states and the District of Columbia, in 2008, 16 states, and in 2010, 20 states used an optional module on anxiety and depression, which included the 8-question Patient Health Questionnaire (PHQ-8) screener for depression, adapted from the 9-item PHQ-9 (9-11), which has a sensitivity of 73% and specificity of 94% for major depression (12). The PHQ-8 omits 1 question on suicidal ideation because interviewers are unable to intervene. We limited all analyses for this report to nonpregnant women of reproductive age (18-44 y) because associations may differ by pregnancy status and BRFSS surveyed too few pregnant women to provide reliable estimates for this group. For 2006, 2008, and 2010, the median state cooperation rates (percentage contacted who completed an interview) were 75% to 76% and the median response rates (percentage eligible for whom an interview was completed) were 51% to 55%. BRFSS has Centers for Disease Control and Prevention institutional review board approval.

We categorized women into 1 of 4 levels of depression status (current major depression, current minor depression, past diagnosis of depression, and no depression) on the basis of their responses to the PHQ-8 screener and 1 additional question assessing whether the woman had ever received a clinical diagnosis of depression. We defined major and minor depression on the basis of responses to the PHQ-8 assessing number of days experiencing anxiety or depression over the last 2 weeks: 1) had little interest or pleasure in doing things; 2) felt down, depressed, or hopeless; 3) had trouble falling asleep; 4) felt tired or had little energy; 5) had a poor appetite or ate too much; 6) felt bad about yourself or that you were a failure; 7) had trouble concentrating on things; and 8) moved or spoke so slowly that other people could have noticed, or the opposite — were so fidgety or restless that you were moving around a lot more than usual. Similar to PHQ-9 methodology (9), we coded major depression as reporting at least 7 days of having little interest or pleasure in doing things or feeling down, depressed, or hopeless and reporting at least 7 days to at least 5 questions total. We coded current minor depression as reporting at least 7 days of having little interest or pleasure in doing things or feeling down, depressed, or hopeless and reporting at least 7 days to 2 to 4 questions total. We further classified the remaining women who did not screen positive for current depression into 2 groups: those with a self-reported previous clinical diagnosis of depression, as an indicator of history of depression, and those with no depression.

Chronic disease conditions and modifiable risk factors inquired of in 2006, 2008, and 2010 included self-reported clinician diagnosis of diabetes or prediabetes, current smoking, binge drinking (>4 drinks at any 1 time) or heavy drinking (>1 drink/d in the past 30 days), overweight (body mass index [BMI] 25.0-29.9 kg/m^2^) or obesity (BMI ≥30.0 kg/m^2^), and physical inactivity (no physical activity in past 30 days aside from regular job).

Initially, we examined differential distribution of demographic characteristics by depression status using χ^2^ tests. Next, we assessed unadjusted associations between depression and 1) diabetes and prediabetes and 2) risk factors for chronic diseases. We also examined the distribution of number of chronic disease conditions and risk factors (0, 1, 2, or ≥3) by depression status using a χ^2^ test. To assess overall odds of 1 or more chronic conditions, in a multivariable multinomial logistic regression model, we calculated the odds ratios (ORs) for 1, 2, and 3 or more chronic conditions and risk factors, compared with 0, among depressed women and those with a past diagnosis of depression compared to women with no depression, adjusted for demographic characteristics. We included demographic characteristics in the multivariable model that, in univariate analyses among all women, were associated with depression status and, in univariate analyses among nondepressed women, were associated with number of chronic conditions and risk factors.

BRFSS surveyed a total of 75,450 women in the states that included the Depression and Anxiety optional module in 2006 (n = 42,444), 2008 (n = 16,261), and 2010 (n = 16,745 women). A total of 69,043 nonpregnant women aged 18 to 44 (91.5%) had information on depression and were included in this report. Of those, 4,895 (7.1%) women did not have information on 1 or more chronic conditions or risk factors, and 5,545 (8.0%) women did not have information on 1 or more demographic characteristics, 93% of whom were missing data on income. Therefore, the regression model included 58,603 (84.9%) women with information on depression status and who were not missing chronic disease or demographic information. We conducted all analyses in SUDAAN (RTI International, Research Triangle Park, North Carolina) to account for the complex sampling design and used weights to produce unbiased prevalence and adjusted odds ratio (AOR) estimates and 95% confidence intervals (CIs).

## Results

Among women of reproductive age, 5.6% met PHQ-8 criteria for major depression, 9.2% met criteria for minor depression, and 13.2% were not currently depressed but had a past diagnosis of depression (Table 1). Additionally, among women with major depression, 67.6% had ever received a clinical diagnosis of depression, and, among women with minor depression, 40.1% had ever received a diagnosis of depression (data not shown). Age, race/ethnicity, education, marital and employment status, and annual household income were associated with depression status.

Compared to women with no depression, prevalence estimates for diabetes and chronic disease risk factors were up to 3.6 times higher among women with major depression, 1.1 to 2.9 times higher for women with minor depression, and 1.1 to 1.9 times higher for women with a past diagnosis of depression (Table 2). The prevalence of 2 and 3 or more chronic conditions and risk factors was significantly higher among women with current depression or a past diagnosis compared to those with no depression (*P* < .001) (Figure). Eighty-nine percent of women who met PHQ-8 criteria for major depression, 85% who met PHQ-8 criteria for minor depression, and 77% with a past diagnosis compared to 66% of nondepressed women had 1 or more chronic conditions or risk factors. Among all women, 12.8% met PHQ-8 criteria for depression and had at least 1 other chronic condition or risk factor (data not shown). The mean number of chronic conditions and risk factors increased with severity of depression (no depression: 2.0; past diagnosis: 2.3; minor: 2.5; major: 2.7).

**Figure.**
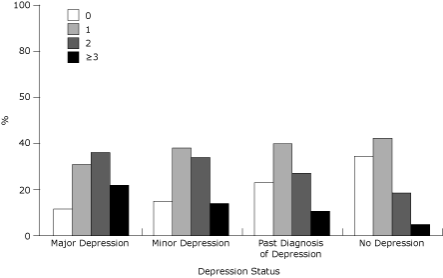
Prevalence and distribution of number of chronic conditions and risk factors by depression status. Conditions and risk factors included diabetes or prediabetes, current smoking, binge drinking or heavy drinking, overweight or obesity, and physical inactivity. χ^2^ *P* < .001. Source: Behavioral Risk Factor Surveillance System, 2006, 2008, and 2010.

**Table:** 

**No. of Chronic Disease Risk Factors**	**Major Depression, %**	**Minor Depression, %**	**Past Diagnosis of Depression, %**	**No Depression, %**
0	11.5	14.8	22.6	34.4
1	30.9	38.1	39.8	42.2
2	35.9	33.3	26.9	18.4
≥3	21.8	13.8	10.6	5.1

In the multivariable, multinomial logistic regression model examining the association between severity and history of depression and number of chronic conditions and risk factors, odds of having at least 3 chronic conditions and risk factors were elevated among women with major (AOR, 5.7; 95% CI, 4.3-7.7), minor (AOR, 4.7; 95% CI, 3.7-6.1), and past diagnosis of depression (AOR, 2.8; 95% CI, 2.4-3.4) (Table 3). Women with current depression or past diagnosis had 1.5 to 3.7 times the odds of having 1 or 2 chronic conditions and risk factors compared to women with no depression. In this sample, 18% of women with 1 or more chronic conditions were currently depressed, compared to 7% of women without a chronic condition or risk factor (χ^2^ *P* < .001) (data not shown).

## Discussion

Almost 13% of US women of reproductive age concurrently experienced both depression and at least 1 additional chronic disease condition or risk factor. Our results suggest a positive dose-response relationship between severity and history of depression and cumulative number of chronic conditions and risk factors, although the temporality of this association is unknown. Both depression and the chronic conditions examined independently place the woman at higher risk of current disease, adverse pregnancy outcomes, and future disease. However, the synergistic effect of poor mental and physical health may have even greater implications for current and future disease risk and future pregnancies.

Associations exist between depression and chronic diseases, such as coronary heart disease (13), cardiovascular disease (4), and diabetes (5), and their risk factors, such as smoking (14,15), alcohol abuse (16), obesity (17), and physical inactivity (18,19) in the general adult population and the elderly. This report shows that associations also exist between depression and diabetes and chronic disease risk factors among women of reproductive age. Of note, associations were not limited to major depression or extremes of chronic conditions, such as diabetes and obesity, but also existed for minor depression and past diagnosis of depression, and precursors to chronic conditions, such as prediabetes and overweight.

Longitudinal research has shown that bidirectional associations exist between chronic conditions and depression through both lifestyle and biologic factors (5,21-25). Because of these common disease pathways, certain interventions, such as exercise (20,21), dietary advice and blood glucose monitoring (22,23), and use of antidepressants (24), may improve a person's physical and mental health.

To prevent or reduce complications from chronic conditions and improve a woman's preconception health, primary care providers and obstetrician/gynecologists often encourage behavior change, such as increasing physical activity, eating a healthier diet, stopping smoking, and reducing alcohol consumption (25). Depressed people may be as motivated to change their behavior as nondepressed people (26). However, behavior change programs may need adaptation to concurrently focus on improving mental health if they are to be successful for depressed people (7,8). Clinicians should consider assessing the mental health of a patient when encouraging lifestyle behavior change, since 18% of women with a chronic condition were currently depressed and these women may have better success with behavior change by simultaneously focusing on mental health improvement.

Past diagnosis of depression was also associated with a higher risk of chronic conditions and risk factors, and research indicates that more than 45% of people with a past depressive episode will relapse (27). Assessing the history of depression may be one way to more fully understand a woman's future risk of chronic disease. Additionally, the increased risk illustrates that treating depression alone may not be sufficient to prevent long-term adverse health outcomes, given that women may have acquired unhealthy behaviors, such as smoking, heavy drinking, or overeating, during a past depressive episode, leaving them at risk of future chronic disease. By assessing lifestyle factors among women with current and past depression, clinicians can refer them to appropriate care when needed.

This report has some limitations. The PHQ-8 is a screener for depression with less than 100% sensitivity and specificity; therefore, some misclassification of current depression status may have occurred. We did not have information on current treatment for depression, and some depressed women currently receiving treatment may not have screened positive with the PHQ-8. A past clinical diagnosis of depression does not capture all women who may have experienced depression in the past but only a limited percentage who may have experienced more severe depression or those with more access to care. Therefore, some women in the "no depression" group may have experienced depression in the past, and any misclassification may have attenuated the association between depression and chronic conditions. Data were self-reported and cross-sectional; we do not know the temporal association between depression and the chronic conditions and risk behaviors. Additionally, other chronic disease risk factors, such as hypertension and high cholesterol, were not assessed in the years examined, when depression was assessed; therefore, the cumulative number of chronic conditions and risk factors are likely conservative estimates. Estimates for past diagnosis of depression and chronic conditions may be underestimated if women were unwilling to report or did not know their status. Additionally, we were unable to examine associations between number, timing, or severity of previous depressive episodes and chronic disease conditions or risk factors.

This study found that more than 1 in 10 reproductive-aged women currently experience depression and have at least 1 additional chronic disease risk factor or condition. Current or previous depression may be an indicator of other chronic conditions and risk factors and, reciprocally, the presence of chronic conditions and risk factors may indicate the woman is at risk for depression. In addition to assessing a woman's physical health, her provider should also consider assessing her mental health when services are available for accurate diagnosis and follow-up (28). This can be done through a brief, 2-item depression screener (29) and asking about history of depression. Detecting and treating poor mental health may prevent or mitigate many chronic conditions and risk factors and may improve a woman's overall and preconception health. In addition, interventions addressing both lifestyle behaviors and depression among reproductive-aged women are needed.

## Figures and Tables

**Table 1:** Demographic Characteristics of Nonpregnant US Women Aged 18 to 44 Years (n = 69,043), by Current Depression Status^a^

**Characteristic**	Depression Status, % (95% CI)				*P* Value^b^

	Major Depression	Minor Depression	Past Diagnosis of Depression	No Depression	
Overall	5.6 (5.1-6.0)	9.2 (8.7-9.8)	13.2 (12.5-13.9)	72.0 (71.1-73.0)	NA
**Age, y**					
18-24	19.5 (17.0-22.2)	26.7 (24.0-29.6)	18.6 (16.9-20.4)	21.1 (20.0-22.4)	.003
25-29	15.6 (13.4, 18.1)	13.9 (12.7, 15.3)	15.9 (14.5, 17.3)	14.9 (14.1, 15.7)	
30-34	19.0 (16.4, 21.9)	19.0 (16.9, 21.3)	19.7 (18.4, 21.1)	20.4 (19.5, 21.2)	
35-39	19.4 (17.3, 21.8)	18.5 (17.0, 20.1)	20.8 (19.4, 22.2)	19.8 (19.0, 20.6)	
40-44	26.5 (24.2, 29.1)	21.9 (19.9, 24.0)	25.2 (23.3, 27.1)	23.9 (22.8, 25.0)	
**Race/ethnicity**					
Black	14.0 (11.4-17.2)	13.5 (11.3-16.0)	5.2 (4.4-6.2)	11.4 (9.8-13.3)	<.001
Hispanic	15.9 (12.4-20.1)	20.3 (15.7-25.8)	13.6 (10.2-18.0)	17.9 (14.0-22.6)	
Other	7.5 (5.9-9.5)	7.9 (6.5-9.4)	4.7 (3.9-5.6)	7.1 (6.3-8.1)	
White	62.6 (58.2-66.9)	58.4 (54.0-62.7)	76.5 (72.5-80.1)	63.6 (59.2-67.7)	
**Education**					
<High school	19.2 (16.4-22.2)	15.1 (12.6-17.9)	8.6 (6.9-10.6)	8.4 (7.0-10.0)	<.001
High school	31.5 (28.3-34.9)	30.2 (27.7-32.8)	24.8 (23.1-26.6)	23.2 (22.1-24.3)	
Some college	33.1 (29.9-36.4)	31.7 (28.8-34.7)	30.2 (28.2-32.2)	29.2 (28.1-30.4)	
College degree	16.3 (13.9-19.0)	23.1 (20.7-25.7)	36.5 (34.5-38.5)	39.2 (37.6-40.9)	
**Marital status**					
Never married	31.4 (28.5-34.5)	30.0 (27.9-32.2)	23.7 (21.9-25.7)	26.4 (25.2-27.6)	<.001
Divorced, separated, or widowed	23.2 (20.9-25.6)	13.1 (11.9-14.5)	13.1 (12.0-14.4)	8.1 (7.6-8.6)	
Unmarried couple	7.4 (6.0-9.1)	8.6 (6.9-10.7)	7.1 (6.1-8.4)	5.6 (4.9-6.4)	
Married	38.0 (35.7-40.5)	48.3 (46.2-50.4)	56.0 (53.7-58.4)	59.9 (58.5-61.3)	
**Employment**					
Employed for wages	42.5 (39.5-45.6)	56.5 (53.6-59.3)	62.8 (60.8-64.7)	64.9 (63.1-66.8)	<.001
Homemaker	13.0 (11.0-15.3)	17.3 (14.7-20.3)	16.8 (15.5-18.2)	17.9 (16.6-19.2)	
Unemployed	17.2 (15.0-19.8)	11.1 (9.6-12.7)	7.2 (6.0-8.7)	5.9 (5.1-6.7)	
Unable to work	17.8 (15.7-20.2)	6.6 (5.3-8.1)	3.9 (3.3-4.6)	1.1 (0.9-1.4)	
Student or retired	9.4 (7.3-12.1)	8.7 (7.4-10.1)	9.4 (8.3-10.6)	10.2 (9.5-10.9)	
**Annual household income, $**					
<15,000	17.5 (15.4-19.7)	8.3 (6.9-9.9)	4.9 (4.1-5.9)	5.2 (4.2-6.4)	<.001
15,000–24,999	37.8 (35.1-40.6)	27.3 (25.4-29.4)	19.4 (17.7-21.2)	17.3 (16.1-18.6)	
25,000–49,999	24.3 (21.5-27.4)	29.8 (27.3-32.4)	26.4 (24.6-28.2)	25.1 (24.0-26.3)	
>50,000	20.4 (17.9-23.2)	34.6 (31.5-37.8)	49.4 (47.0-51.8)	52.4 (50.2-54.6)	

Abbreviations: CI, confidence interval; NA, not applicable.

a Current depression defined based on PHQ-8, adapted from PHQ-9 (9-11); major depression defined as reporting ≥7 d of having little interest or pleasure in doing things or feeling down, depressed, or hopeless and reporting ≥7 d to ≥5 questions total. Minor depression defined as reporting ≥7 d of having little interest or pleasure in doing things or feeling down, depressed, or hopeless and reporting ≥7 d to 2-4 questions total; past diagnosis based on self-report. Source: Behavioral Risk Factor Surveillance System, 2006, 2008, and 2010.

b Calculated by using χ^2^ test.

**Table 2:** Prevalence of Diabetes and Chronic Disease Risk Factors by Current Depression Status^a^ Among Nonpregnant US Women Aged 18 to 44 Years (n = 69,043)

**Characteristic**	Depression Status, % (95% CI)				*P* Value^b^

	Major Depression	Minor Depression	Past Diagnosis of Depression	No Depression	
**Diabetes status**					
Prediabetes	1.8 (1.3-2.5)	0.9 (0.6-1.3)	0.9 (0.6-1.4)	0.5 (0.4-0.6)	<.001
Diabetes	6.4 (5.3-7.6)	5.3 (4.3-6.4)	3.5 (2.9-4.3)	1.8 (1.6-2.1)	
**Current smoker**					
Yes	44.1 (40.5-47.7)	29.6 (27.6-31.8)	26.8 (24.7-29.0)	14.2 (13.2-15.4)	<.001
**Alcohol consumption^c^**					
Binge or heavy drinker	17.6 (15.2-20.4)	17.8 (15.9-19.9)	19.7 (18.5-20.9)	14.5 (13.8-15.2)	<.001
**BMI^d^**					
Overweight	24.7 (22.5-27.1)	28.7 (26.1-31.5)	26.9 (25.1-28.8)	25.5 (24.5-26.5)	<.001
Obese	35.8 (32.3-39.4)	34.9 (32.8-37.1)	28.2 (26.2-30.2)	19.9 (19.1-20.9)	
**Physical activity**					
Inactive^e^	41.6 (38.4-44.8)	31.5 (29.3-33.8)	21.2 (19.3-23.2)	18.9 (18.0-19.9)	<.001

Abbreviations: CI, confidence interval; BMI, body mass index.

a Current depression defined based on PHQ-8, adapted from PHQ-9 (9-11); major depression defined as reporting ≥7 d of having little interest or pleasure in doing things or feeling down, depressed, or hopeless and reporting ≥7 d to ≥5 questions total. Minor depression defined as reporting ≥7 d of having little interest or pleasure in doing things or feeling down, depressed, or hopeless and reporting ≥7 d to 2-4 questions total; past diagnosis based on self-report. Source: Behavioral Risk Factor Surveillance System, 2006, 2008, and 2010.

b Calculated by using χ^2^ test.

c Heavy drinker defined as >1 drink/d and binge drinker defined as >4 drinks on 1 occasion in the past 30 days.

d Overweight defined as BMI 25.0-29.9 kg/m^2^; obese defined as BMI ≥30.0 kg/m^2^.

e No physical activity other than normal job in past 30 days.

**Table 3:** Association Between Depression and Number of Chronic Conditions and Risk Factors^a^ Among Nonpregnant US Women Aged 18 to 44 Years (n = 58,603)

Characteristic^b^	No. of Chronic Conditions and Risk Factors, AOR (95% CI)		

	1	2	≥3
Major depression	1.7 (1.3-2.2)	3.7 (2.8-4.8)	5.7 (4.3-7.7)
Minor depression	1.9 (1.5-2.3)	3.5 (2.8-4.3)	4.7 (3.7-6.1)
Past diagnosis of depression	1.5 (1.3-1.7)	2.2 (1.9-2.5)	2.8 (2.4-3.4)
No depression	1 [Reference]		

Abbreviations: AOR, adjusted odds ratio; CI, confidence interval.

a Defined as diabetes or prediabetes, smoking, overweight or obesity, heavy drinking or binge drinking, and physical inactivity.

b Current depression defined based on PHQ-8, adapted from PHQ-9 (9-11); major depression defined as reporting ≥7 d of having little interest or pleasure in doing things or feeling down, depressed, or hopeless and reporting ≥7 d to ≥5 questions total. Minor depression defined as reporting ≥7 d of having little interest or pleasure in doing things or feeling down, depressed, or hopeless and reporting ≥7 d to 2-4 questions total; past diagnosis based on self-report. Source: Behavioral Risk Factor Surveillance System, 2006, 2008, and 2010.

## References

[1] Farr SL, Bitsko RH, Hayes DK, Dietz PM (2010). Mental health and access to services among US women of reproductive age. Am J Obstet Gynecol.

[2] Strine TW, Mokdad AH, Dube SR, Balluz LS, Gonzalez O, Berry JT (2008). The association of depression and anxiety with obesity and unhealthy behaviors among community-dwelling US adults. Gen Hosp Psychiatry.

[3] Goodwin RD, Davidson KW, Keyes K (2009). Mental disorders and cardiovascular disease among adults in the United States. J Psychiatr Res.

[4] Van der Kooy K, van Hout H, Marwijk H, Marten H, Stehouwer C, Beekman A (2007). Depression and the risk for cardiovascular diseases: systematic review and meta analysis. Int J Geriatr Psychiatry.

[5] Mezuk B, Eaton WW, Albrecht S, Golden SH (2008). Depression and type 2 diabetes over the lifespan: a meta-analysis. Diabetes Care.

[6] Yonkers KA, Wisner KL, Stewart DE, Oberlander TF, Dell DL, Stotland N (2009). The management of depression during pregnancy: a report from the American Psychiatric Association and the American College of Obstetricians and Gynecologists. Gen Hosp Psychiatry.

[7] Fagerstrom K, Aubin HJ (2009). Management of smoking cessation in patients with psychiatric disorders. Curr Med Res Opin.

[8] van der Merwe MT (2007). Psychological correlates of obesity in women. Int J Obes (Lond).

[9] Kroenke K, Spitzer RL, Williams JB (2001). The PHQ-9: validity of a brief depression severity measure. J Gen Intern Med.

[10] (1994). Diagnostic and statistical manual of mental disorders.

[11] (1994). Diagnostic and statistical manual of mental disorders.

[12] Spitzer RL, Kroenke K, Williams JB (1999). Validation and utility of a self-report version of PRIME-MD: the PHQ primary care study. Primary Care Evaluation of Mental Disorders. Patient Health Questionnaire. JAMA.

[13] Rugulies R (2002). Depression as a predictor for coronary heart disease. A review and meta-analysis. Am J Prev Med.

[14] Boden JM, Fergusson DM, Horwood LJ (2010). Cigarette smoking and depression: tests of causal linkages using a longitudinal birth cohort. Br J Psychiatry.

[15] Quattrocki E, Baird A, Yurgelun-Todd D (2000). Biological aspects of the link between smoking and depression. Harv Rev Psychiatry.

[16] Conner KR, Pinquart M, Gamble SA (2009). Meta-analysis of depression and substance use among individuals with alcohol use disorders. J Subst Abuse Treat.

[17] Luppino FS, de Wit LM, Bouvy PF, Stijnen T, Cuijpers P, Penninx BW (2010). Overweight, obesity, and depression: a systematic review and meta-analysis of longitudinal studies. Arch Gen Psychiatry.

[18] Roshanaei-Moghaddam B, Katon WJ, Russo J (2009). The longitudinal effects of depression on physical activity. Gen Hosp Psychiatry.

[19] Teychenne M, Ball K, Salmon J (2008). Physical activity and likelihood of depression in adults: a review. Prev Med.

[20] Mead GE, Morley W, Campbell P, Greig CA, McMurdo M, Lawlor DA (2009). Exercise for depression. Cochrane Database Syst Rev.

[21] Rethorst CD, Wipfli BM, Landers DM (2009). The antidepressive effects of exercise: a meta-analysis of randomized trials. Sports Med.

[22] Crowther CA, Hiller JE, Moss JR, McPhee AJ, Jeffries WS, Robinson JS (2005). Effect of treatment of gestational diabetes mellitus on pregnancy outcomes. N Engl J Med.

[23] Ismail K, Winkley K, Rabe-Hesketh S (2004). Systematic review and meta-analysis of randomised controlled trials of psychological interventions to improve glycaemic control in patients with type 2 diabetes. Lancet.

[24] Hughes JR, Stead LF, Lancaster T (2003). Antidepressants for smoking cessation. Cochrane Database Syst Rev.

[25] Johnson K, Posner SF, Biermann J, Cordero JF, Atrash HK, Parker CS, CDC/ATSDR Preconception Care Work Group (2006). Recommendations to improve preconception health and health care — United States: a report of the CDC/ATSDR Preconception Care Work Group and the Select Panel on Preconception Care. MMWR Recomm Rep.

[26] Siru R, Hulse GK, Tait RJ (2009). Assessing motivation to quit smoking in people with mental illness: a review. Addiction.

[27] Eaton WW, Anthony JC, Gallo J, Cai G, Tien A, Romanoski A (1997). Natural history of Diagnostic Interview Schedule/DSM-IV major depression. The Baltimore Epidemiologic Catchment Area follow-up. Arch Gen Psychiatry.

[28] (2009). Screening for depression in adults: US preventive services task force recommendation statement. Ann Intern Med.

[29] Kroenke K, Spitzer RL, Williams JB (2003). The Patient Health Questionnaire-2: validity of a 2-item depression screener. Med Care.

